# Exploring Vitamin E’s Role in Colorectal Cancer Growth Using Rodent Models: A Scoping Review

**DOI:** 10.3390/nu18020289

**Published:** 2026-01-16

**Authors:** Nuraqila Mohd Murshid, Jo Aan Goon, Khaizurin Tajul Arifin

**Affiliations:** 1 Department of Biochemistry, Faculty of Medicine, Universiti Kebangsaan Malaysia, Cheras, Kuala Lumpur 56000, Malaysia; nuraqilamohdmurshid@ukm.edu.my (N.M.M.); joaan@ukm.edu.my (J.A.G.); 2 Ageing and Degenerative Diseases Research Group, Universiti Kebangsaan Malaysia, Bangi 43600, Malaysia

**Keywords:** carcinoma, carcinogenesis, foci, gavage, polyp

## Abstract

**Background:** Vitamin E has been studied for its role in reducing the growth of colorectal cancer (CRC). CRC is a worldwide health concern. A meta-analysis reported that CRC patients have a lower concentration of serum vitamin E, suggesting it to be a risk factor. Although rodent models are widely used in disease research, their application in studying vitamin E as a preventive or therapeutic agent in CRC is not well characterized. To address this gap, we conducted a scoping review to examine the available evidence, adhering to the PRISMA-ScR checklist. **Methods**: We searched PubMed, Google Scholar, Scopus, and Web of Science (WoS) for full-text English original articles published before May 2024, using Medical Subject Headings (MeSH) terms and free text. The following search string strategy was applied: (Vitamin E OR tocopherol$ OR tocotrienol$) AND (Colo$ cancer OR colo$ carcinoma) AND (Rodentia OR mouse OR Rodent$ OR mice OR murine OR rats OR guinea OR rabbit OR hamsters OR Animal model OR Animal testing OR animals) AND (neoplasm$ OR “tumor mass” OR tumor volume OR tumor weight OR tumor burden). Data were charted into five categories using a standardized, pretested form. The charted data were synthesized using descriptive and narrative methods. **Conclusions**: This study highlights that γ- and δ-tocopherols, as well as δ-tocotrienol and its metabolites, were reported to reduce tumor volume and formation in various rodent models. While these results are promising, this scoping review identifies a need for further research to address translational barriers such as dosing, bioavailability, and long-term safety before clinical application.

## 1. Introduction

Cancer is an aggressive disease that contributes to high mortality. According to GLOBOCAN 2022, colorectal cancer incidence is placed 4th, and 3rd for mortality, worldwide for both genders and all ages [1,2,3]. This disease impacts a country economically (screening, diagnosis, treatment, and palliative care), and psychologically, for patients and loved ones [4,5].

Colorectal cancer (CRC) typically initiates with the benign proliferation of mucosal epithelial cells [6], forming growths known as polyps [7]. These polyps can develop and enlarge gradually for 10 to 20 years before they progress to cancerous lesions [8]. Uncontrolled growth that extends to the wall of the colon [9] can penetrate the blood or lymphatic vessels, resulting in metastasis to adjacent lymph nodes or even to distant organs [10]. CRC is often diagnosed at a late stage, significantly impairing treatment outcomes and survival rates. Current treatments encompass a range of strategies, including surgery [11], chemotherapy, and radiation therapies [12]. By prioritizing prevention and early detection, the incidence and mortality of CRC can be markedly reduced.

There is a growing interest in exploring the potential of natural products as complementary or alternative treatments for CRC. Published research has provided evidence that certain natural compounds can reduce tumor size and inhibit cancer progression in animals [13,14,15] and in cell cultures [16,17]. Vitamin E is an antioxidant and signaling molecule involved in the scavenging of free radicals and a reduction in oxidative stress [18]. The isomers of this vitamin namely, tocopherols and tocotrienols, have widely been reported to exhibit positive effects on the prevention of multiple disorders and conditions such as Alzheimer’s disease [19], myocardial infarction [20], Parkinson’s disease [21,22], muscle [23] and bone degeneration [24,25], and preeclampsia [26]. With the presence of a chromanol ring and isoprenoid side chain, this vitamin scavenges free radicals by reducing them into stable molecules and, in turn, the vitamin itself becomes a tocopheroxyl radical which later forms stable dimers or is reduced by other antioxidants [27]. As a signaling molecule, vitamin E influences the activity of enzymes and receptors by modulating the expression of genes in crucial pathways, such as the immune response, metabolism of toxins and xenobiotics, mitosis, synaptic transmission, apoptosis, and many kinases-regulated signaling pathways [28].

The strong correlation between vitamin E and redox balance has promoted its use in cancer prevention. Scientists believe vitamin E may prevent cancer, become an adjunct therapy for the disease, and delay cancer progression [29]. Although tocopherols positively affected age-associated disorders, the α- and γ-tocopherol isomers showed lower anticancer activity than the δ in lung tumorigenesis in mice [30]. The authors of a review paper suggested future cancer prevention trials consider using mixtures of isomers, such as those containing γ- and δ-, to reap the benefits of each isomer [31].

What if vitamin E causes cancer? A genome-wide association study (GWAS) on more than 290,000 cancer cases calculated the potential causal effect of circulating vitamin E on the risk of ten common cancers, including colorectal cancer. The study concluded there was no definite link between the level of circulating vitamin E and cancer occurrence [32]. A meta-analysis reported that CRC patients generally have a lower concentration of serum vitamin E. Hence, reduced serum vitamin E levels were suggested as a risk factor for CRC [33].

Animal models, particularly rodents, are widely used in colorectal cancer (CRC) research because they allow for investigation of tumor initiation, progression, immune modulation, and systemic metabolic effects within an intact organism. Compared with in vitro systems, rodent models provide a more integrated biological context, enabling assessment of tumor–microenvironment interactions, immune responses, and the pharmacokinetic and pharmacodynamic behavior of compounds such as vitamin E. While rodent models do not fully recapitulate human CRC due to interspecies differences in metabolism, gastrointestinal physiology, and microbiota, these limitations are well-recognized in translational research. The primary motivation for this scoping review was to map the extent and characteristics of studies specifically examining vitamin E in rodent CRC models, a gap not previously addressed.

Accordingly, our research questions were framed to reflect this aim: (i) Which rodent models have been employed in CRC studies involving vitamin E? (ii) Which forms of vitamin E have been investigated and how have their effects been reported in preclinical settings? The intention was not to infer direct clinical applicability or to rank vitamin E isoforms for human use, but rather to characterize the preclinical evidence base, identify research patterns, and highlight knowledge gaps.

## 2. Materials and Methods

### 2.1. Literature Source and Study Selection

The final protocol was prospectively registered with the Open Science Framework on 19 November 2024 (https://osf.io/69sau, accessed on 23 September 2025). We separately searched PubMed, Google Scholar, Scopus, and Web of Science (WoS) for original, full-text, English articles published until May 2024, using Medical Subject Headings (MeSH) terms and free text. Terms were searched in all fields. The following search string strategy was applied: (Vitamin E OR tocopherol$ OR tocotrienol$) AND (Colo$ cancer OR colo$ carcinoma) AND (Rodentia OR mouse OR Rodent$ OR mice OR murine OR rats OR guinea OR rabbit OR hamsters OR Animal model OR Animal testing OR animals) AND (neoplasm$ OR “tumor mass” OR tumor volume OR tumor weight OR tumor burden).

### 2.2. Inclusion Criteria

Two reviewers screened the titles and abstracts compiled from the four databases in Rayyan, an online application. We included studies that (1) investigated tumor growth in rodents, (2) used vitamin E as a standalone supplement, (3) assessed tumor growth, (4) used animals from the Rodentia order, and (5) were original research articles. Disagreements between researchers concerning article selection were discussed with a third reviewer and consensus was reached.

### 2.3. Exclusion Criteria

Studies were excluded if they were (1) published in languages other than English, (2) using vitamin E as part of a mixture or a vehicle, or (3) reporting insufficient data to be included in this review.

### 2.4. Data Items and Data Abstraction Process

Data were abstracted into the following categories: animal model, vitamin E characteristics, tumor metrics, conditions of intervention, and results. A standardized Excel form, developed a priori and tested on four studies, was used for data abstraction to ensure clarity and consistency. In line with the purpose of the scoping reviews, critical appraisal of individual sources was not performed, as the objective was to map the scope and characteristics of the existing evidence. The results are presented in tables and descriptive text. This review was conducted by adhering to the Preferred Reporting Items for Systematic reviews and Meta-Analyses extension for Scoping Reviews (PRISMA-ScR) checklist [34].

## 3. Results

**Figure 1:**
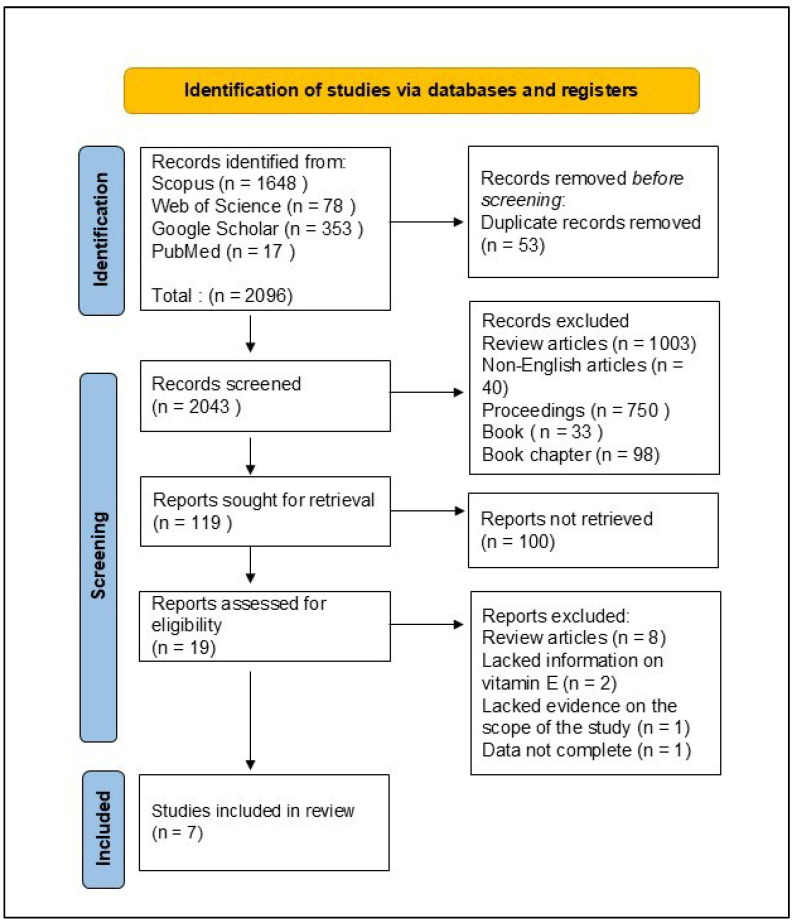
Flowchart of the literature search and article selection.

The search in the four databases generated 2043 publications (Figure 1). Duplicates were removed, followed by non-original research articles, leaving 14 articles to be screened. Of these, seven were excluded due to irrelevant supplements or cancer types, leaving seven studies that met the inclusion criteria and were subjected to full-text analysis.

The seven studies summarized in Table 1 utilized different rodent models, predominantly mice, with two studies involving rats [18,35]. The studies explored the impact of tocopherols and tocotrienols on tumor number, size, volume, and weight. The interventions involved dietary supplementation, oral gavage, and intraperitoneal injection of vitamin E compounds, with some studies initiating supplementation before and after cancer induction.

As summarized in Table 1, four studies investigated the effects of tocopherols on CRC in rodents [35,36,37,38]. The number of tumors was assessed by counting the total number of aberrant crypt foci (ACF) and aberrant crypts (ACs) under a microscope [35] and counting the number of macroscopic tumors [37]. Tumor size was represented as mm^2^ by calculating the area of the tumor [37], while tumor volume was calculated using the mathematical formula of a sphere: tumor volume = 4/3πr3 (r = average radius of tumor) [36]. One study weighed the mass (mg) of the tumors [38].

Most of the interventions produced positive results, whereby γ-tocopherol (γTF) [37], δ-tocopherol (δTF) and α-tocopherol mixture (γTmt), reduced the number of tumors in mice [36] and rats [35], respectively. α-Tocopheryl succinate (TS) significantly reduced the weight of tumors in mice [38].

These studies consistently demonstrated that γ- and δ-tocopherols significantly reduced the number of tumors in rodent models (Table 1). Notably, α-tocopherol showed the least efficacy in reducing tumor growth [35,36]. These two studies supplemented tocopherols into the animals’ diets. The only study that used a form of α-tocopherol as a curative measure introduced TS intraperitoneally, in solution and also with the aid of nanoparticles [38]. The intraperitoneal delivery resulted in significant inhibition of tumor growth in the mouse model.

Three studies used tocotrienols in their interventions by introducing them into the diet either via natural feeding or by oral gavage (Table 2). Tumor growth was assessed by the number of tumors [18,39] and weight of tumors [15]. One study classified tumors into two size categories—small (<2 mm^2^) and large (>2 mm^2^)—then quantified the number of tumors in each category [39].

## 4. Discussion

This scoping review evaluates the potential role of vitamin E in modulating tumor growth in rodent models of CRC. Animal models, particularly rodents, are widely used in CRC research because they enable the investigation of tumor initiation, progression, immune modulation, and systemic metabolic effects within an intact organism. Compared with in vitro systems, rodent models provide a more integrated biological context, enabling assessment of tumor–microenvironment interactions and the pharmacokinetic behavior of vitamin E. Although rodent models do not fully reflect human colorectal cancer due to species-specific differences in metabolism and gastrointestinal physiology, these limitations are well recognized and inherent to translational cancer research. The findings indicate that specific isomers of vitamin E exert significant antitumor effects in these models. However, the variability in responses based on the form of vitamin E used, timing of intervention, and variation in the methods highlights the complexity of vitamin E’s role. Current evidence predominantly supports the chemopreventive rather than therapeutic efficacy of vitamin E isoforms, suggesting they are more plausibly positioned as adjunctive agents in established tumors rather than primary treatments.

The studies included in this review provide strong evidence that γ- and δ-tocopherols significantly reduced the number of tumors, especially before and during carcinogen treatments [35,36], but not the volume. γ-TmT was also effective in reducing the number of tumors [35]. However, 0.1% γ-tocopherol failed to reduce tumor count when supplementation was performed before the induction of three cycles of dextran sodium sulfate (DSS) as compared to fewer cycles of DSS [36]. Vitamin E potentially inhibits tumor progression by quenching free radicals, the induction of cell differentiation, cell cycle inhibition, or the induction of apoptosis [29], as well as by inducing the elimination of tumor cells by the immune system [40,41,42]. However, these effects can be overwhelming with immense progression in the growth of tumor cells [37].

α-Tocopherol was ineffective in all studies, except one [38] using α-tocopheryl succinate (TS). The methyl group on the chromanol ring of α-tocopherol probably deters it from quenching reactive oxygen and nitrogen species effectively as compared to unmethylated counterparts in δ- and γ-tocopherols [36]. TS was also evidenced as a strong inducer of apoptosis, unlike α-tocopherol [43].

The use of TS as a treatment was prominent compared to other interventions, as it was incorporated intraperitoneally as a curative method [38], rather than being used as a preventive approach [35,36,37]. This finding is corroborated by previous studies that documented TS alone [44] and in synergy with another antioxidant [45] as reducing the tumor volume in mice breast cancer models.

The triumph of the TS regimen is attributed to the binding action of TS to the ubiquinone site (succinate dehydrogenase) in Complex II of the mitochondria in cancer cells. The inhibition of the enzyme causes electrons to leak, leading to surplus generation of reactive oxygen species (ROS), ultimately triggering apoptosis [46]. TS activity has been compared with that of α-tocopherol, and the former exhibited a stronger antitumor effect by activating apoptosis via Apo2 ligand while specifically being non-toxic to normal cells [47].

Despite these promising results in terms of tumor number reduction, the studies present inconsistent findings regarding tumor volume. For instance, Chen et al. [36] noted no significant changes in tumor volume, even though δ- and γ-tocopherols reduced the number of tumors. This suggests that tocopherols may be more effective at preventing the initiation of tumorigeneses rather than influencing the growth of already established tumors. The lack of significant tumor volume emphasizes the need for further exploration of tocopherol’s role at different stages of cancer progression.

Of the studies that were examined, three addressed the effects of tocotrienols delivered via dietary approaches or oral gavage. All analyzed tocotrienol variants exhibited significant antitumor properties. Previous studies have demonstrated that fisetin, statins, capecitabine, and green tea polyphenols similarly reduce carcinogenesis, indicating possible synergistic benefits when used with tocotrienols [48,49,50,51,52].

Preclinical findings emphasized the effective chemopreventive properties of δ-tocotrienol (DT3). Husain et al. [18] discovered that DT3 decreased polyp formation by 70% and incidence of cancer by 99% over a 20-week treatment program. This efficacy exceeded that of sulindac, an established chemopreventive drug. In parallel, recent meta-analyses indicate that other agents, including resveratrol, demonstrate notable anticancer properties, highlighting the necessity for multifaceted strategies in CRC prevention [53]. Additionally, in a recent study, δ-tocotrienol combined with aspirin significantly reduced colon cancer cell viability and suppressed cancer stem cell markers, highlighting its potential for targeted therapy [18].

Jang et al. [39] revealed that δTE-13′-COOH reduced the number of tumors by inhibiting pro-inflammatory enzymes, inducing apoptosis and autophagy in cancer cells, by regulating their sphingolipid pathways. Similarly, numerous studies indicate that the modulation of the NF-κB pathway, which is implicated in inflammation, plays a crucial role in colorectal cancer progression. This suggests that a comprehensive approach targeting multiple pathways could enhance therapeutic efficacy [54]. While these findings are promising, further clinical studies are essential to establish the therapeutic potential and safety of δ-tocotrienol in human populations. Additionally, plenty of research highlights the ongoing exploration of novel combinatorial therapies that integrate tocotrienols with established treatments for enhanced efficacy in CRC management [55].

In addition to the effects of DT3, palm oil-derived TRF also demonstrated significant anticancer properties. These effects are particularly mediated through the inhibition of the Wnt signaling pathway, which plays a critical role in colorectal tumorigenesis. For instance, Zhang et al. [15] showed that TRF effectively suppressed tumor growth in Balb/C nude mice by reducing Wnt3a expression and preventing the nuclear translocation of β-catenin. This pathway, which is commonly dysregulated in CRC, presents a valuable therapeutic target. Other studies support these findings, showing that inhibitors of the Wnt pathway, such as IWP-2, significantly reduce colorectal cancer cell proliferation, further reinforcing the therapeutic potential of TRF in targeting this pathway [56,57].

The studies included in this review used different chemicals to induce cancer. Most of the studies treated the animals with vitamin E before the induction of cancer [15,35,36,37], mainly to test the chemopreventive effect of vitamin E. Studies to determine the effect of vitamin E after the development of cancer in animal models are limited, most likely because the progression of cancer is different in every animal, thus making it difficult to assess the exact stage of cancer at which treatment with the vitamin should begin.

One of the discrepancies in the studies is the method of administering vitamin E; via gavage [15], orally [18], transported in nanoparticles injected intraperitoneally (Hama et al., 2022 [38]), and through the incorporation of vitamin E in the animals’ diet [35,36,37,39]. Intraperitoneal injection allowed for the delivery of chemicals directly to the targeted spot [38]. Oral gavage is the administration of the test compounds to the animals’ stomachs via the esophagus using a gavage needle. This technique enables researchers exact control over the timing and dosage of the test compound [58], but is known to induce stress in the animals [59], and may affect their behavior and metabolism [58].

Providing the animals with a diet containing the test compound directly mimics the human experience of food. However, due to the rapid oxidation of vitamin E [60], maintaining its stability in the animal diet is a concern. Studies that mixed vitamin E with the animal diet [35,36,37] stated that the diets were stored at 4 °C until use, except for one study that did not mention how the diet was stored [39]. These concerns could be eliminated if the stability of the vitamin E was verified at the end of the experiment [61].

In general, the supplementation of tocotrienol and tocopherol to the animals significantly reduced the growth of tumors. While these findings are promising, the effective management of CRC encompasses a multidisciplinary approach, including prevention, early detection, diagnosis, treatment, and follow-up, as outlined in the Malaysian Clinical Practice Guidelines [62]. Recent studies by Cenin et al. [63] suggest that personalized screening protocols based on genetic risk factors may improve early detection rates. As personalized medicine continues to evolve, there is potential for tailored treatment protocols that integrate tocotrienols with other therapies based on individual patient risk factors and tumor characteristics. This approach can increase detection rates, reduce unnecessary procedures, improve patient outcomes, optimize resource allocation, and promote patient engagement, more effective treatment, and ultimately, improved survival rates for CRC patients [64].

Despite promising preclinical results in both in vitro and in vivo models from this evaluation of the available literature until May 2024, a notable gap exists in translating these findings into clinical practice. While preclinical studies such as those by Husain et al. [18] demonstrated promising results in animal models, further human clinical trials are needed to establish the effectiveness of δ-tocotrienol in CRC prevention and treatment. Many preclinical studies were conducted on animal models, which do not always reflect human biology, raising concerns about the generalizability of the results. Therefore, future clinical trials should focus on larger, diverse patient populations and explore the long-term efficacy and safety of tocotrienols in humans.

Additionally, variability in vitamin E formulations, dosages, and administration routes complicates the comparison of outcomes across studies. This complication was reiterated by the lack of uniformity in measuring the outcomes of the research (tumor number, tumor size, etc.). Furthermore, most existing research needs long-term follow-up data to assess the sustained efficacy and safety of tocotrienols in diverse patient populations. As a result, there is a critical need for well-designed, randomized controlled trials to evaluate the therapeutic potential of tocotrienols in humans, including optimal dosing strategies, treatment regimens, and possible interactions with standard chemotherapy agents. Such studies will not only clarify the role of tocotrienols in colorectal cancer management but also contribute to the establishment of evidence-based guidelines for their clinical application.

Future clinical trials are crucial to establish the optimal dosing, bioavailability, and therapeutic regimens of tocotrienols for patients with CRC. Additionally, it is important to investigate the potential synergistic effects of tocotrienols when combined with standard treatments such as chemotherapy and targeted therapies, as this could enhance treatment outcomes and reduce side effects.

Our review has certain limitations. We restricted our search to studies published in the English language, which may have led to language bias and the exclusion of relevant evidence reported in other languages. In addition, only four databases (PubMed, Scopus, Web of Science, and Google Scholar) were searched. Although these are widely used and cover a substantial body of the literature, studies indexed in other databases or unpublished sources may have been missed, thereby limiting the comprehensiveness of our evidence base.

## 5. Conclusions

Nevertheless, despite the limitations, the reviewed studies consistently demonstrated that vitamin E effectively reduces tumor number and size in rodent models of CRC. These findings suggest that vitamin E has potential as a chemopreventive agent for CRC, particularly when administered before or during the early stages of carcinogenesis. However, variations in experimental designs and delivery methods complicate direct comparisons. Further research, including well-designed human trials, is needed to establish optimal dosing strategies, therapeutic regimens, and long-term safety to translate these promising preclinical findings into clinical practice.

## Figures and Tables

**Table 1:** Comparative analysis of tocopherol intervention in rodent models.

Animal Model	Vitamin E Characteristics	Tumor Metrics	Condition of Intervention	Results	Study
5-week-old male and female hCYP1A mice [Cyp1a2/Cyp1a1tm2Dwn Tg (CYP1A1, CYP1A2)1Dwn/DwnJ and C57BL/6J]	0.2% of δ-, γ-, or α-TF to AIN-93M diet	i. Volume (mm^3^); V = 4/3πr3 ii. Number of tumors	Preventive: δ-TF-, γ-TF- and α-TF-supplemented diet one week before PhIP administration.	Reduction in the number of tumors in δ-TF (↓↓↓ 64%) and γ-TF (↓ 45%) groups, no significant changes in α-TF group. No changes in tumor volume. No changes in female mice.	Chen et al. [36]
			Preventive: δ-TF-supplemented diet for one week, followed by a complete DSS treatment, then fed with control diet	At 10 weeks after PhIP administration, ↓ 44% reduction in tumor multiplicity. No changes in female mice.	
			Preventive: control diet at first before switching to the 0.2% δ-T-supplemented diet immediately after finishing the DSS treatment.	No changes in tumor volume (mm^3^).	
			δ-TF supplementation before or after PhIP and DSS treatment,	No changes in tumor volume in both male and female mice.	
Male Balb/c mice (5–6 weeks)	γTF and mixed TF (45% γTF, 45% δTF and 10% (+)-α- TF acetate)-supplemented AIN-93G diet at 0.1% diet, respectively.	i. Tumor size: large (>2 mm^2^) and small (<2 mm^2^) ii. Number of tumors	Preventive: γ-TF and mixed tocopherol one week before AOM and three cycles of DSS (1.5–2.5%) treatment.	No changes.	Jiang et al. [37]
			Preventive: γ-TF and mixed TF one week before AOM and 1.5% DSS treatment.	γ-TF ↓ reduced the number of large-sized tumors/mouse; no significant changes with mixed TF.	
			Curative: γ-TF three days after tumor induction with AOM and two cycles of 1.5% DSS injection.	Γ-TF ↓ reduced number of large-sized (>2 mm^2^) tumors/mouse.	
7-week-old male F344 Rats	0.2% of δ-, γ-, α- or a γ-TF rich mixture of tocopherols (γ-TmT), (tocopherols) γ-TmT, containing 57% γ-TF, 24% δ-TF, 13% α-TF, and 1.5% β-TF, in diet	Numbers of aberrant crypt foci (ACF)	Preventive: One week before AOM injection.	δ-TF, γ-TF and γ-TmT ↓ number of ACF/rat. No changes in α-TF group.	Guan et al. [35]
Male Hos:HR-1 hairless and BALB/cCrSlc mice	50 mM of TS	Number of tumors	Curative: 50 mM of TS contained in egg phosphatidylcholine (EPC) as TS-NP nanoparticles and TS solution intraperitoneally, 5- and 8 days post cancer induction, through injection with colon26-Luc cells.	↓ inhibited tumor growth for both TS-NP and TS treatments.	Hama et al. [38]

↓ = significant reduction *p* < 0.05; ↓↓↓ = significant reduction *p* < 0.001; AOM = azoxymethane; ACF = aberrant crypt foci; DSS = dextran sodium sulfate; PhIP = 2-amino-1-methyl-6-phenylimidazo[4;5-b] pyridine; TF = tocopherol; TS = α-tocopheryl succinate.

**Table 2:** Comparative analysis of tocotrienol intervention in rodent models.

Animal Model	Vitamin E Characteristics	Tumor Metrics	Condition of Intervention	Results	Study
Male Balb/c mice (5–6 weeks old	δTT-13′-COOH (metabolite of δ-TT (0.022% in diet)	i. Tumor size: large (>2 mm^2^) and small (<2 mm^2^) ii. Number of tumors	Curative: One week after induction of cancer by AOM/DSS.	↓↓ tumor numbers for both small (<2 mm^2^) and large size (>2 mm^2^) categories. ↓ number of large-sized tumors by 58%.	Jang et al. [39]
Male (17–22 g) and female (14–19 g) BALB/c nude mice (6–8 weeks old)	5, 10 and 20 mg/kg body weight of TRF by gavage	Tumor weight	Preventive: Two weeks before tumor induction.	↓ tumor weight (g/mouse) for both males and females for 5, 10, and 20 mg/kg body weight of TRF.	Zhang et al. [15]
Female Fish- er 344 rats (6 weeks old, 120–140 g)	δ-TT (200 mg/kg orally twice a day) for 20 weeks	i. Number of tumors ii. Number of polyps	Curative: Supplemented after initiating cancer with AOM (15 mg/kg, s.c.).	↓↓↓ tumor formation. ↓ polyps formation.	Husain et al. [18]

↓ = significant reduction *p* < 0.05; ↓↓ = significant reduction *p* < 0.005; ↓↓↓ = significant reduction *p* < 0.001; AOM = azoxymethane; DSS = dextran sodium sulfate; TRF = tocotrienol-rich fraction; TT = tocotrienol.

## Data Availability

The original contributions presented in this study are included in the article. Further inquiries can be directed to the corresponding author.

## Abbreviations

The following abbreviations are used in this manuscript:

CRC	Colorectal cancer
ACF	Aberrant crypt foci
ACs	Aberrant crypts
AOM	Azoxymethane
DSS	Dextran sodium sulfate
TRF	Tocotrienol-rich fraction
TT	Tocotrienol
PhIP	2-amino-1-methyl-6-phenylimidazo[4,5-b] pyridine
TF	Tocopherol
GWAS	Genome-wide association study
PRISMA-ScR	Preferred Reporting Items for Systematic reviews and Meta-Analyses extension for Scoping Reviews.
γTF	γ-tocopherol
δTF	δ-tocopherol
γTmt	α-tocopherol mixture
TS	α-Tocopheryl succinate
WoS	Web of Science
MESH	Medical Subject Headings
